# First-pass stress perfusion MR Imaging findings of apical hypertrophic cardiomyopathy: with relation to LV wall thickness and late Gadolinium-enhancement

**DOI:** 10.1186/1532-429X-17-S1-Q66

**Published:** 2015-02-03

**Authors:** Jin Young Yoo, Eun Ju Chun, Yeo-Koon Kim, Sang Il Choi

**Affiliations:** 1 Radiology, Seoul National University Bundang Hospital, Seongnam-si, Korea (the Republic of; 2 Radiology, Kangbuk Samsung Hospital, Seoul, Korea (the Republic of

## Background

To evaluate the prevalence and pattern of perfusion defect (PD) on first-pass stress perfusion MR imaging in relation with the degree of left ventricular hypertrophy (LVH) and late gadolinium-enhancement (LGE) in patients with apical hypertrophic cardiomyopathy (APH).

## Methods

Cardiac MR imaging with first-pass stress perfusion, cine, and LGE sequence was performed in 26 patients with APH from January 2008 to December 2012. We analyzed a total of 416 segments for LV wall thickness on end-diastolic phase of cine images, and evaluated the number of hypertrophied segment and number of consecutive hypertrophied segment (NCH). We assessed the presence or absence of PD and LGE from all patients. If there was PD, we subdivided the pattern into sporadic (sporadic-PD) or ring (ring-PD). Using univariate logistic method, we obtained the independent predictor for presence of overall PD and ring-PD.

## Results

PD on stress perfusion MRI was observed in 20 patients (76.9%), 12 of them (60%) showed ring-PD. Maximal LV wall thickness and number of hypertrophied segment were independent predictors for overall PD (all, p < 0.05). NCH with more than 3 segments was an additional independent factor for ring-PD. However, LGE was not statistically related with PD in patients with APH.

## Conclusions

About three quarters of the patients with APH showed PD, most of them represented as ring-PD. LVH degree or distribution was related with pattern of PD, however, LGE was not related with PD. Therefore, the clinical significance of PD in the patients with APH seems to be different from those with non-APH, and further comparison study between the two groups should be carried out.

## Funding

N/A.

**Figure 1:**
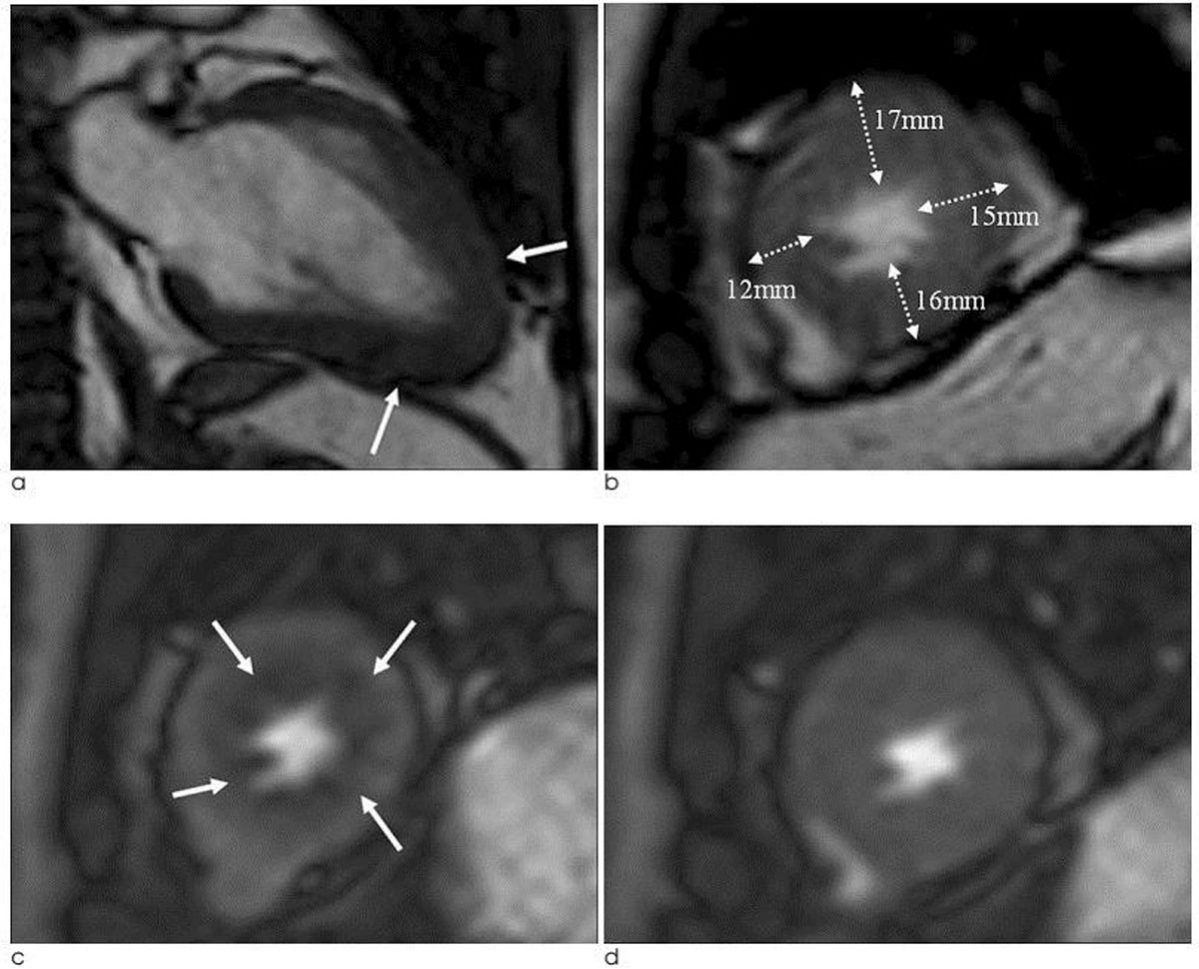
A 45-year old male patients with angina pain who visited to emergency room was confirmed as apical HCM by typical ECG and echocardiographic findings, and he performed CMR for risk stratification. a. Two-chamber cine image shows apical wall thickening with typical ‘spade of ace' sign (arrows). b. Short-axis cine image shows LVH at apical anterior, lateral and inferior wall (NHS of 3, NCH-3). c, d. First-pass stress perfusion (c) and rest perfusion (d) show reversible ring of subendocardial perfusion defect (arrows) at whole apical layer.

**Table 1:** Independent Predictors for Perfusion Defect

	For overall perfusion defect		For ring of subendocardial perfusion defect					
		95% CI			95% CI			

	HR	upper	lower	p-value	HR	upper	lower	p-value

Age (years)	0.98	0.90	1.08	0.983	1.00	0.92	1.08	0.960

Sex	0.80	0.07	8.91	0.856	4.40	0.42	46.26	0.217

BMI (kg/m^2^)	1.11	0.73	1.69	0.633	1.18	0.81	1.72	0.378

Hypertension	1.50	0.21	10.82	0.688	1.20	0.21	6.88	0.838

Diabetes	0.01	0.00	-	0.999	2.60	0.21	32.90	0.461

Current smoker	0.64	0.09	4.89	0.643	0.53	0.10	2.98	0.474

Hypercholesterolemia	0.86	0.12	6.01	0.877	6.00	0.92	39.19	0.061

Family history of sudden cardiac death or HCM	0.10	0.01	1.49	0.095	5.00	0.04	6.55	0.597

Med_aspirin	0.33	0.05	2.21	0.255	1.25	0.24	6.63	0.793

Med_antithrombotic drug	0.01	0.00	-	1.000	0.01	0.00	-	1.000

Symptom	0.75	0.11	5.11	0.769	0.78	0.16	3.80	0.756

Typical angina including syncope	3.18	0.30	33.26	0.334	6.60	0.97	44.93	0.054

Ejection Fraction (%)	0.99	0.86	1.14	0.883	1.08	0.95	1.22	0.260

LV mass (g)	1.01	0.99	1.03	0.322	1.01	1.00	1.02	0.203

Maximal LV wall thickness (mm)	1.97	1.05	2.43	0.029*	1.50	1.06	2.13	0.021*

LGE	3.00	0.45	19.93	0.255	3.75	0.59	23.87	0.162

NHS	1.82	1.01	3.30	0.048*	1.40	0.99	1.97	0.056

AHS-2	5.67	0.75	42.58	0.092	2.00	0.30	13.51	0.477

AHS-3	3.71	0.54	25.59	0.183	9.00	1.39	58.44	0.021*

Note. - LGE = late gadolinium-enhancement, NHS = Number of hypertrophied segment, AHS-2 = more than 2 adjacent hypertrophied segments, AHS-3 = more than 3 adjacent hypertrophied segments

* p < 0.05

